# A review of human exposure to PFAS: substantial contribution from seafood

**DOI:** 10.1265/ehpm.25-00002

**Published:** 2025-09-19

**Authors:** Yukiko Fujii, Kouji H. Harada

**Affiliations:** 1 Department of Pharmaceutical Sciences, Daiichi University of Pharmacy, 22-1 Tamagawa-machi, Minami-ku, Fukuoka 815-8511, Japan; 2 Laboratory of Food Hygiene and Environmental Health, Kyoto Prefectural University, Shimogamo, Sakyo, Kyoto 606-8522, Japan

**Keywords:** Per- and polyfluoroalkyl substances, Perfluorooctanoic acid, Perfluorooctane sulfonic acid, Perfluoroalkyl carboxylic acid, Fish, Tap water, Food, Seafood

## Abstract

Per- and polyfluoroalkyl substances (PFAS) have recently been shown to affect human health at low levels in the blood, according to epidemiological evidence. Consequently, human exposure to these chemicals should be strictly controlled to prevent health risks. This review reports on the potential sources of PFAS using Japan as an example. Tap water has attracted attention as a source of exposure to PFAS. PFAS have also been detected in the air, in household dust, and in consumer products. Furthermore, in the general population, diet is the most common source of exposure, and there is particular concern about human exposure to PFAS accumulated in seafood. Continuous monitoring is important for appropriate management of exposure for both humans and the environment.

## Graphical Abstract

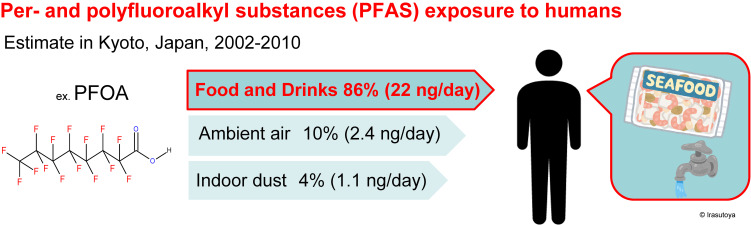

## 1. Background

Per- and polyfluoroalkyl substances are abbreviated as PFAS. This group of compounds has stable methyl or methylene groups, in which the hydrogen atoms are replaced by fluorine atoms (perfluoroalkyl chains, e.g. CF_3_-, -(CF_2_)*_n_*-, and CF_3_-(CF_2_)*_n_*-). Several thousand PFAS are currently in practical use. This chemical group undergoes degradation and metabolism in parts other than the perfluoroalkyl chain, which produces extremely persistent perfluoroalkyl carboxylic acids (PFCAs) or perfluoroalkyl sulphonic acids (PFSAs) as end products that are thought to remain in the environment. Both PFCAs and PFSAs have a variety of analogs with different carbon numbers. For example, the PFCA with eight carbons is called perfluorooctanoic acid (PFOA) and the PFSA with the eight carbons is called perfluorooctane sulfonic acid (PFOS). Moreover, PFCAs with more than eight carbons are called long-chain PFCAs (LC-PFCAs). Epidemiological evidence has shown that PFAS affect human health through immunosuppression and developmental effects, even at low serum concentrations [1]. Recently, the Japan Environment and Children’s Study reported the possibility of a link between maternal PFAS exposure and chromosomal abnormalities [2]. Consequently, human exposure to these chemicals should be strictly controlled to prevent health risks. Although the toxicities of PFSA and their exposure sources have been reviewed [3, 4], there are still many unclear points in the PFAS exposure pathway. This review reports on the potential sources of exposure using the case of Japan as an example.

## 2. Contamination of exposure media with PFAS

The general population is indirectly exposed to environmental chemicals, while workers who handle PFAS suffer from direct exposure (occupational exposure). This section outlines indirect exposure to PFAS for the general population. Indirect exposure is the uptake of chemicals through exposure media (e.g., air, beverages, and food) after chemicals are released into the environment (e.g., air, soil, and the aquatic environment). Indirect exposure also includes exposure through the use of consumer products containing these chemicals.

### 2.1. Contributions of exposure pathways in the general population

In Japan, PFAS contamination of groundwater and tap water has been found in several areas and is of concern for exposure of humans to these chemicals. PFAS have also been found in many products. For example, cosmetics and sunscreens contained high concentrations of PFAS at parts-per-million levels at maximum [5], and recycled plastic pellets had low concentrations of PFAS at parts-per-trillion levels in average [6]. Humans are likely exposed to PFAS in household dust after use of the products. In addition, previous studies have indicated that diet is the main source of PFAS exposure for humans [7, 8].

To clarify the situation in the 2000s, when the PFAS problem became apparent in Japan, we estimated the contribution of each pathway to PFOA exposure (Table 1). The PFOA concentrations in duplicate diet samples (all food and drink consumed in 24 h) in Kyoto, Japan, in 2009 [8], in household dust in 2010 [9], and in ambient air in 2002 [10] were used. The estimated adult intake of indoor dust is 50 mg/day [11] and adult humans inspire 13.3 m^3^ of air/day, with 69% of the particles in air respirable and PFOA completely absorbed into the body. The largest estimated exposure is through duplicate diet (86% of the total intake), followed by ambient air (10%), and indoor dust (4%). These results suggest that dietary intake (duplicate diet) is the major source of PFOA exposure for humans.

**Table 1:** Adult exposure to perfluorooctanoic acid (PFOA) in Kyoto, Japan.

**Sample**	**PFOA Intake^a^ (ng/day)**	**Contribution to total (%)**	**Sampling date**	**Reference**
Duplicate diet^b^	21.8	86.2	December 2009	Fujii et al., 2012 [8]
Indoor dust	1.1^c^	4.3	December 2010	Liu et al., 2011 [9]
Ambient air	2.4^d^	9.5	March 2002	Harada et al., 2005 [10]
Total	25.3			

^a^Calculated from the geometric mean of the PFOA concentrations.

^b^All food and drink consumed in 24 h.

^c^Estimated assuming that the adult human intake of indoor dust was 50 mg/day.

^d^Estimated assuming that the adult human intake of air was 13.3 m^3^/day and that 69% of the particles in air were respirable.

### 2.2 Contamination of tap water

**Fig. 1:**
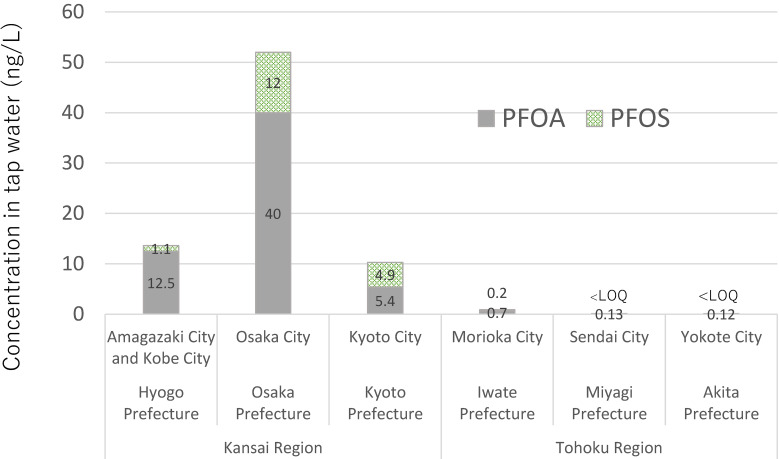
PFOA and PFOS in tap water from Japan in 2003 (Saito et al., 2004) [13].

PFOS and PFOA have been detected in high concentrations in groundwater. Contaminants in soil spread to the aquatic environment and affect groundwater and drinking water. Both PFOS and PFOA are difficult to remove in water purification processes (coagulation, sedimentation, filtration, and chlorine disinfection processes). In a previous study, the concentrations of PFOS and PFOA in tap water using subsoil water as a source were the same as in river water affected by effluents from water treatment plants [12]. As an example of regional contamination, Saito et al. reported that the concentration of PFOA in drinking water sampled in 2003 was much higher in Osaka in the Kansai region of Japan, than in other areas of Japan [13] (Fig. 1). Furthermore, in 2016 in Okinawa Prefecture, the Okinawa Prefectural Bureau of Public Enterprises, which operates the drinking water treatment plant, announced that PFOS contamination was present in the tap water and unprocessed water (raw water for tap water) at the intake source of the Chatan Water Filtration Plant [14]. Follow-up investigations and countermeasures are required.

### 2.3 Pollution of river waters

Rivers are frequently contaminated with PFAS. In Japan, a survey of PFOS and PFOA in rivers at 79 sites in Japan was reported in 2003 [13]. PFOA concentrations were highest in the Tama River (31.4 ng/L) in the Kanto region of Japan, and highest in the Yodo (141 ng/L) and Ina (Kanzaki) (456 ng/L) Rivers in the Yodo river system in the Kansai region of Japan. According to a 2007 survey conducted by Osaka Prefecture, the Kansai region of Japan, 600 ng/L of PFOA was detected in the Ai River within the Kanzaki River basin [15]. A fluoropolymer manufacturing factory (Daikin Industries Ltd) is located near the Ai River, and the company has disclosed that PFOA was manufactured and used at the factory until 2012 [16]. Osaka Prefecture has been continuously monitoring the public waterways and groundwater around the factory. The 2024 survey detected PFOA levels of up to 5,700 ng/L in nearby waterways and up to 30,000 ng/L in the groundwater [17]. This factory is thought to be a major source of PFOA emissions in this area. Moreover, it has been suggested that contaminants in treated effluent from sewage treatment plants originate from industrial additives containing PFOA and PFOS. In addition, fire extinguishing agents containing PFOS are used at airports and released in effluent. The Yodo river system was surveyed again in 2013 and PFCAs other than PFOA, with carbon chain lengths that are shorter than eight, were also detected [18].

### 2.4 Contamination of seafood

**Fig. 2:**
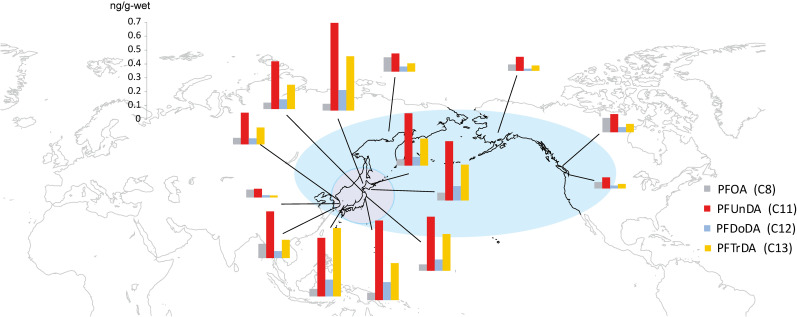
PFOA and LC-PFCAs in the edible parts (muscle) of Pacific cod from the North Pacific. Sampling was conducted from 2015 to 2017. This figure is reprinted from Fujii et al. 2019 [19], Copyright (2019), with permission from Elsevier.

The pollution of rivers (Section 2.3) may result in PFAS run-off into the sea around Japan and contamination of edible seafood. A monitoring study of edible Pacific cod in the North Pacific Ocean revealed that PFCAs, especially LC-PFCAs, were detected at high concentrations in cod from waters around Japan [19, 20] (Fig. 2). In some cases, estimated intakes based on daily seafood intake have closely agreed with total dietary intake [19]. A tendency for blood PFAS concentrations to increase with seafood intake has been observed in the Japanese population [21–23]. Compared with residents of other countries, residents of Japan consume more seafood and are more likely to be exposed to environmental chemicals such as PFAS that have bioaccumulated in the marine ecosystem. Studies measuring PFAS in the blood of Japanese people have reported that LC-PFCAs are present at higher concentrations [24, 25] than in blood from residents of other countries, including the US [26], Germany [27], Sweden [28], and Vietnam [25]. Seafood consumption is thought to contribute to the high blood concentrations of PFCAs in Japan.

**Fig. 3:**
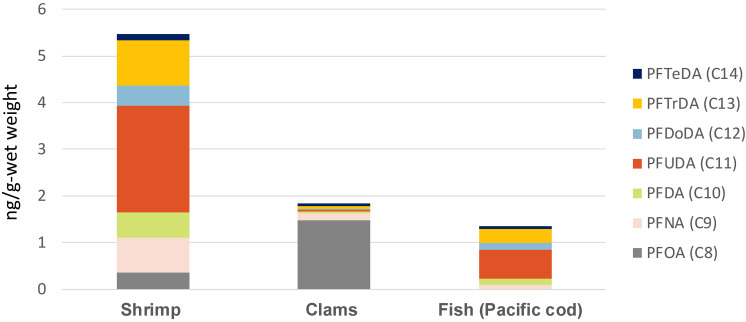
Comparison of PFCA concentrations in edible shrimp, clams, and Pacific cod from Pacific coast of Japan. The results are all from the edible parts (whole body for from Suruga Bay (Fujii et al., 2024) [30], everything except the shell for clams from Aichi (Fujii et al., 2020) [29], and muscle for Pacific cod from off-Miyagi (Fujii et al., 2019) [19]). The seafood samples were purchased from local retail outlets.

Further studies of seafood, such as clams [29] and shrimp [30], have revealed high concentrations of PFCAs. Recent studies have shown that PFAS have different bioaccumulation potentials from the general fat solubilities indicated by the octanol/water partition coefficients for legacy persistent organic pollutants (POPs), such as polychlorinated biphenyls and dioxins. For example, cases have been reported where the amounts of PFCAs in clams and shrimps (lower trophic levels) are higher than those in cod (higher trophic levels), and there are large differences in the composition of PFCAs detected in these organisms [30] (Fig. 3). Investigation of the dynamics of PFAS in aquatic environments and the mechanisms of bioaccumulation in marine biota are needed to assess the sources of PFAS exposure to humans in detail.

## 3. Recommended values for PFAS in drinking water and food in the United States, the European Union, and Japan

The provisional lifetime health advisory (LHA) values for tap water published by the United States (US) Environmental Protection Agency (EPA) in 2009 were 400 ng/L PFOA and 200 ng/L PFOS. In 2016, the first established LHA value for PFOA and PFOS combined was greatly reduced to 70 ng/L [31]. This new value was established using developmental toxicity results from animal studies [32]. Furthermore, in 2022, much lower provisional revised LHA values of 0.004 ng/L for PFOA and 0.02 ng/L for PFOS were set [31]. This change was made after considering risk assessments from various epidemiological studies and was indexed to suppression of antibody titers following combination vaccination (diphtheria and tetanus) of children. Although these LHA values are currently difficult to achieve for most drinking water, PFAS exposure should be reduced as much as possible. In 2023, a Class 1 drinking water regulation value of 4 ng/L was set for each of PFOA and PFOS [31], and this was made official in April 2024 for full implementation from 2029 (extended to 2031 by Trump administration in May 2025). In 2020, the Japanese Ministry of Health, Labour and Welfare set a provisional water quality control target of 50 ng/L for PFOA and PFOS combined using the same reference dose as the 2016 LHA value in the US [33]. This 50 ng/L value is also used as the provisional guideline for environmental water by the Japanese Ministry of the Environment. From April 2026, the regulatory limits of 50 ng/L for tap waters will be enacted under Water Supply Act. The European Union has drinking water directives of 100 ng/L for the sum of the concentrations of 20 PFAS and 500 ng/L for the total concentration of PFAS [34].

The European Food Safety Authority (EFSA) established a tolerable weekly intake (TWI) of 4.4 ng/kg bw/week for sum of PFOS, PFOA, perfluorononanoic acid (PFNA), and perfluorohexanesulfonic acid (PFHxS) in 2020 [35]. Based on this TWI, maximum levels (the upper limit) for PFOA PFOS, PFNA, and PFHxS were set in 11 animal products, including seafood, in 2022. The maximum level for PFOA in crustaceans and bivalve mollusks was set at 0.7 µg/kg wet weight [36]. Using PFOA levels in clams [29] purchased in Japan in 2017 that we analyzed in an earlier study, some samples were exceed this limit. In 2024, the Japanese Food Safety Commission adopted the 2016 US EPA risk assessment and set a tolerable daily intake (TDI) of 20 ng/kg bw/day for PFOA and PFOS [37]. In this case, PFAS intake from seafood would not exceed the TDI. Currently, the risk assessment criteria used in different countries vary, and in-depth risk assessments of PFAS are required to establish common criteria.

## 4. Conclusions

Exposure to a wide range of PFAS occurs in the general population. Serum concentrations of bioaccumulative PFAS are associated with seafood intake. The Japanese population may have a higher baseline PFAS exposure. This is due to their high seafood intake. Health effects are a concern for PFAS, and further studies are needed to determine whether adverse effects occur. POP designation under the Stockholm Convention was applied to PFOS in 2009, PFOA in 2019, PFHxS in 2022, and LC-PFCAs in 2025. In the future, it will be necessary to monitor whether regulation of the production of these chemicals has reduced human exposure. It is expected that the population exposed to high concentrations from consumer product sources will reduce over time after regulation. However, PFAS that have already been released may remain in the environment, and these compounds may continue to be present in tap water and food. It is possible that exposure will be difficult to reduce even after regulation. If PFAS are still detected at high concentrations in the environment and exposure media after regulation, some countermeasures to reduce their concentrations will be necessary. Moreover, there are many unknowns about the vast majority of PFAS, which have not been targeted by regulations to date, with regard to residues in the environment, human exposure, and health effects. Continuous monitoring is important for appropriate management of exposure, and it is necessary to consider both human and environmental perspectives.

## Footnotes

This article is based on research of Y.F. that received the Young Investigator Award from the Japanese Society for Hygiene and its associated lecture at the 93rd annual meeting of the Japanese Society for Hygiene held in Tokyo, Japan, on 2–4 March 2022.

## Abbreviations

EPA: Environmental Protection Agency
LC-PFCA: long-chain perfluoroalkyl carboxylic acid
LHA: lifetime health advisory
PCBs: polychlorinated biphenyls
PFAS: Per- and Polyfluoroalkyl substances
PFCAs: perfluoroalkyl carboxylic acids
PFHxS: perfluorohexanesulfonic acid
PFNA: perfluorononanoic acid
PFOA: perfluorooctanoic acid
PFOS: perfluorooctane sulfonic acid
PFSAs: perfluoroalkyl sulphonic acids
POPs: persistent organic pollutants
US: United States

## Declarations

### Availability of data and materials

The data that support the findings of this study are available from the corresponding author upon reasonable request.
